# Successful ureteral stent placement with rendezvous technique for ureteral obstruction after urinary diversion: A case report

**DOI:** 10.1016/j.radcr.2024.07.079

**Published:** 2024-08-14

**Authors:** Mariko Irizato, Yozo Sato, Shinichi Murata, Shohei Chatani, Akira Ouchi, Takashi Kinoshita, Hidekazu Yamaura, Yoshitaka Inaba

**Affiliations:** a Department of Diagnostic and Interventional Radiology, Aichi Cancer Center Hospital, Aichi, Japan; b Department of Radiology, Shiga University of Medical Science, Otsu, Japan; c Department of Gastroenterological Surgery, Aichi Cancer Center Hospital, Aichi, Japan

**Keywords:** Postoperative ureteral complications, Urinary diversion, Rendezvous technique, Ureteral stent placement

## Abstract

Ureteral obstruction after urinary diversion is not a rare complication, and the treatment is generally the ureteral stent placement via antegrade approach via the nephrostomy. We present a case of 64-year-old man with history of total pelvic resection and urinary diversion for local recurrence of rectal cancer who presented bilateral ureteral obstruction due to postoperative adhesion. First, bilateral nephrostomies were performed. The antegrade approach via nephrostomy could not break through the obstruction in the left side. Therefore, antegrade and retrograde approaches were attempted, and the internal-external drainage catheter could be placed by the rendezvous technique using bilateral microcatheters and microguidewires. The patient was able to avoid a permanent nephrostomy and continues to undergo regular internal drainage catheter exchange. Permanent nephrostomy considerably reduces the patient's quality of life, and in cases of tight obstruction, rendezvous techniques can be used.

## Introduction

A postoperative ureteral complication (PUC) is a potential complication of any abdominal or pelvic surgery, including gynecological surgery, urinary diversion to an ileal conduit, and colorectal surgery [1].

Although treatment of these PUCs generally includes ureteral stent placement and surgical repair, surgical repair is often difficult because of postoperative adhesions. Percutaneous nephrostomy is another treatment option; however, long-term external drainage leads to deterioration of the patient's quality of life.

For PUC after urinary diversion, ureteral stent placement with cystoscopic retrograde approach is sometimes difficult and an antegrade approach via nephrostomy is used. However, when these approaches fail, the combination with antegrade and retrograde approach, so-called rendezvous technique, is required.

The following case report describes ureteral stent placement using the rendezvous technique with coaxial system in patient with ureteral tight obstruction after urinary diversion.

## Case presentation

A 64-year-old man, 2 years and 6 months after rectal amputation for rectal cancer (cT2N0M0), was diagnosed with local recurrence with invasion into the prostate. He underwent total pelvic resection and urinary diversion with ileal conduit. His postoperative course was good and bilateral ureteral stents were removed; however, he developed a urinary tract infection early afterwards. Bilateral nephrostomy was promptly performed to avoid delaying the introduction of postoperative adjuvant chemotherapy. Reinsertion of ureteral stents through the ileal conduit was considered; however, nephrostomy seemed optimal for reducing the risk of infection during subsequent chemotherapy. Postoperative chemotherapy was performed with the external drainage. After completion of chemotherapy, reestablishment of an internal drainage was attempted to improve the patient's quality of life.

**Fig. 1:**
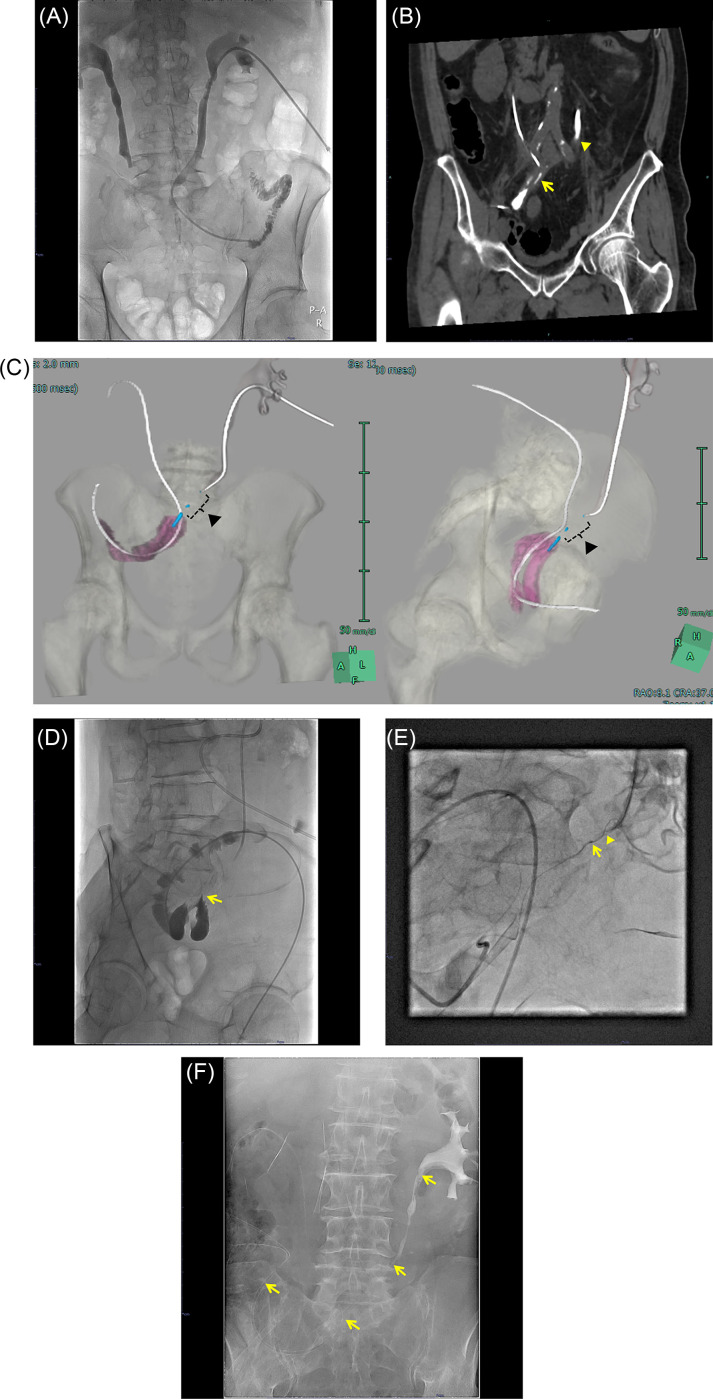
A 64-year-old man after total pelvic resection and urinary diversion with ileal conduit. He developed a postoperative urinary tract infection and a nephrostomy was constructed. (A) Bilateral nephrostomies were created owing to urinary tract infection. The right side was successfully converted to an internal-external drainage via the antegrade approach; however, the left side was failed. (B) Loopgraphy-CT image showed the anastomotic site of the left ureter and nearby ureter (arrows), and the obstructed portion of the left ureter (arrowheads). (C) The reconstructed image shows the ileal conduit (pink), ureteral anastomosis and nearby ureter (blue), and the tip of the catheter inserted through the left nephrostomy. The length of the obstructed portion was relatively long (arrowheads). (D) Based on Loopgrapgy-CT, contrast from the ileal conduit identified the left ureteral anastomosis (arrow). (E) The obstruction was broken through the rendezvous technique using microcatheters and microguidewires from both sides of the nephrostomy and ileal conduit. The microcatheter tip from ileal conduit (arrow) and penetration by microguidewire (arrowhead). (F) After establishing the pull-through route, a 6Fr internal-external drainage catheter (arrows) was successfully inserted.

Loopography from the nephrostomy did not depict the ileal conduit, and both ureters were obstructed. An antegrade approach was attempted to advance the guidewire through the obstruction using a 6.5 Fr seeking catheter (Hanako Medical, Saitama, Japan) and 0.035-inch Radifocus guidewire (Terumo, Tokyo, Japan); however, it failed. Another day, the right side was successfully converted to an internal–external drainage by supporting using a 7Fr introducer sheath (Terumo, Tokyo, Japan). However, in the left side, advancing the guidewire with the same system was failed owing to strictly obstruction than the right side (Fig. 1A). To determine the location of the left ureter and ileal conduit suture, CT image was taken with contrast medium injection from the nephrostomy and the ileal conduit (Loopography-CT). Loopography-CT image showed the anastomotic site of the left ureter and ileal conduit, and the obstructed portion of the left ureter (Fig. 1B). The length of the obstructed portion was relatively long and the ileal conduit was bent nearby ureteral anastomosis (Fig. 1C). According to these findings, the ureteral obstruction was suspected to be owing to postoperative adhesion, which resulted in ureteral torsion and chronic urinary tract infection.

The procedure was decided after a multidisciplinary discussion among gastroenterological surgeons, urologists, and interventional radiologists. After obtaining informed consent, including the advantages of avoiding a permanent left-sided nephrostomy and the potential risks of perforation, urinoma, and ureteral-arterial fistula, the internal–external drainage catheter placement using the rendezvous technique via antegrade and retrograde approaches was attempted. By forming the 6.5Fr seeking catheter in the Shepherds–Hook shape, the left ureteral anastomosis was successfully selected from the ileal conduit side (Fig. 1D). At first, breaking through the obstructed potion using the 1.7Fr microcatheter (Carnelian Pixie; Tokai Medical Products, Aichi, Japan) and 0.016-inch microguidewire (GT wire; Terumo, Tokyo, Japan) via the antegrade route was tried, but failed. Next, the 1.5Fr microcatheter (Veloute Ultra; Asahi Intecc, Aichi, Japan) and 0.014-inch micro-guidewire (Meister; Asahi Intecc, Aichi, Japan) were used via the retrograde route and breaking through the obstructed portion was successfully performed (Fig. 1E). Subsequently, the snare catheter was inserted via the antegrade route, and microguidewire via the retrograde route was caught by snare catheter. After establishing a pull-through route, a 6Fr straight catheter as an internal–external drainage was finally inserted (Fig. 1F). There were no major complications in the procedure. At a later date, bilateral internal–external drainage catheters were changed to bilateral single J catheters as an internal drainage. The periodic catheter exchanges have been continued during 4 years follow-up after procedure.

## Discussion

Ureteral stenosis after ileal conduit urinary diversion occurs mainly at the anastomosis and can occur in 1.3% to 10% of patients [2]. It is related to fibrosis and scarring related with ischemia and urinary leakage [3,4]. In particular, on the left side, urinary stenosis can occur away from the anastomosis owing to the long segment of ureteral dissection and the excessive angulation of the ureter across the mesentery of the sigmoid mesentery [5,6]. In the present case, the patient developed a urinary tract infection relatively early after operation. Although a nephrostomy was performed, the patient subsequently developed an obstruction in the distal ureter, suggesting that inflammation was the cause. The ileal conduit was bent and the ureter was twisted, which may have contributed to the obstruction.

Treatment varies depending on the site of obstruction, and ureteral stent placement is often attempted first. Although good results have been reported for surgical procedures [7], the patient in the present case had undergone 2 previous surgeries, and reoperation is considered difficult. Because a percutaneous nephrostomy had already been performed for urinary tract infection, percutaneous antegrade ureteral stenting was considered first. If the severe stenosis makes the usual antegrade approach difficult, the rendezvous technique with antegrade and retrograde approaches may be considered. Kawada et al. [8] in 2020 reported a high technical success rate of 17 of 19 cases for urinary stenting using the rendezvous technique, although only 2 patients with ileal conduits were included. The retrograde approach via the ileal conduit is considered more difficult than that via the bladder because the ileal conduit is tortuous and the ureteral orifice is not physiologically structured. Herein, the left ureteral anastomosis was successfully selected by creating a 6.5Fr seeking catheter with a Shepherds Hook shape via the ileal conduit. In previous reports, a technique using a 5Fr catheter and a hydrophilic guidewire was used to break through the stenosis [8,9]. However, in this case, a coaxial system was used because of severe stenosis. To the best of our knowledge, this is the first report on ureteral stent placement using the rendezvous technique with a coaxial system in a patient with an ileal conduit.

According to Kawada et al [8] in *2020*, none of the 6 patients who rendezvoused at the retroperitoneal cavity owing to major leakage achieved a stent-free status. This suggests that route epithelialization is unexpected when rendezvoused at the retroperitoneal cavity. In the present case, the possibility of breaking through outside the ureter cannot be ruled out because the complete obstruction was penetrated using a guide wire. Therefore, stent-free is considered difficult; however, the patient's quality of life was greatly improved by avoiding a permanent nephrostomy.

Several possible complications of the internal and external drainage exist using the rendezvous technique. In the antegrade approach, ureteral injury and urinoma formation may occur. Iliac artery fistula is a rare but serious complication, especially in patients with tissue Inflammation after surgery or radiation therapy [10]. The use of a stiff wire with high penetrating force may lead to serious complications. Therefore, we should correctly confirm the site of occlusion and carefully perform the procedure. Hence, loopography-CT with concurrent forward and retrograde contrast to identify the site of occlusion was performed, and it was able to confirm that the occlusion was distal to the intersection with the iliac artery. Furthermore, a coaxial system that may prevent the penetration of the wire into the retroperitoneum was used. Regarding the retrograde approach, perforation of the ileal conduit or injury of the ureteral anastomosis can occur, especially in the early postoperative period.

## Conclusion

In conclusion, we have reported a case of ureteral stent placement using the rendezvous technique with a coaxial system in patient with ureteral tight obstruction after urinary diversion. Permanent nephrostomy greatly reduces the patient's quality of life, and in cases of tight obstruction, rendezvous techniques can be a considerable approach.

## Patient consent

Informed consent for the publication of this case report was obtained from the patient.
